# Supplementary material for: CTLA4-Linked Autoimmunity in the Pathogenesis of Endometriosis and Related Infertility: A Systematic Review
Source: Int J Mol Sci. 2022 Sep 18;23(18):10902. doi: 10.3390/ijms231810902 (PMC9504308; doi:10.3390/ijms231810902)
Supplement: Supplementary file 1 [file ijms-23-10902-s001.zip › ijms-1900929-supplementary.pdf]

Supplementary Table S1: List of articles (PMID) excluded from the literature review

	PMID	Exclusion criteria
1	34656305	Not including CTLA-4
2	34647523	Not including CTLA-4
3	33947339	Not including CTLA-4
4	33953208	Not including CTLA-4
5	33428059	Not including CTLA-4
6	33078970	Not including CTLA-4
7	32711226	Not including CTLA-4
8	31993049	Not including CTLA-4
9	30784825	Not including CTLA-4
10	30325236	Not including CTLA-4
11	30595667	Not including CTLA-4
12	29990511	Review
13	29845209	Not including CTLA-4
14	29234882	Not including CTLA-4
15	26959830	Not including CTLA-4
16	25764161	Not including CTLA-4
17	25367742	Not including CTLA-4
18	22687138	Not including endometriosis
19	22652893	Review
20	20146732	Not including CTLA-4
21	19300397	Not including CTLA-4
22	18314122	Not including CTLA-4
23	18405309	Review
24	17953947	Not including CTLA-4
25	17509578	Not including CTLA-4
26	18292821	Not including CTLA-4
27	17118166	Review
28	16311290	Not including CTLA-4
29	12447413	Review
30	11287041	Not including CTLA-4
31	10333368	Review
32	35404426	Not including CTLA-4
33	34865852	Not including CTLA-4
34	32334621	Not including CTLA-4
35	32231038	Not including CTLA-4
36	30447298	Not including CTLA-4
37	27488034	Not including CTLA-4
38	21508908	Not including CTLA-4

39	20732991	Not including CTLA-4
40	15831297	Not including CTLA-4
41	35628346	Review
42	34945174	Review
43	34367125	Not including anti-CTLA-4
44	34039410	Not including CTLA-4
45	33505394	Review
46	33278837	Review
47	33147452	Not including CTLA-4
48	32751735	Not including CTLA-4
49	32666129	Not including CTLA-4
50	32280445	Not including CTLA-4
51	31985569	Review
52	31540116	Not including CTLA-4
53	31418859	Not including CTLA-4
54	34829910	Not including CTLA-4
55	34501240	Not including CTLA-4
56	34345285	Not including CTLA-4
57	34103260	Not including CTLA-4
58	33411080	Review
59	33209739	Not including CTLA-4
60	32933042	Not including CTLA-4
61	32698242	Not including CTLA-4
62	32311852	Not including CTLA-4
63	31869309	Not including CTLA-4
64	31418859	Not including CTLA-4
65	31099938	Not including CTLA-4
66	30826860	Review
67	30684339	Not including endometriosis
68	30367890	Not including CTLA-4
